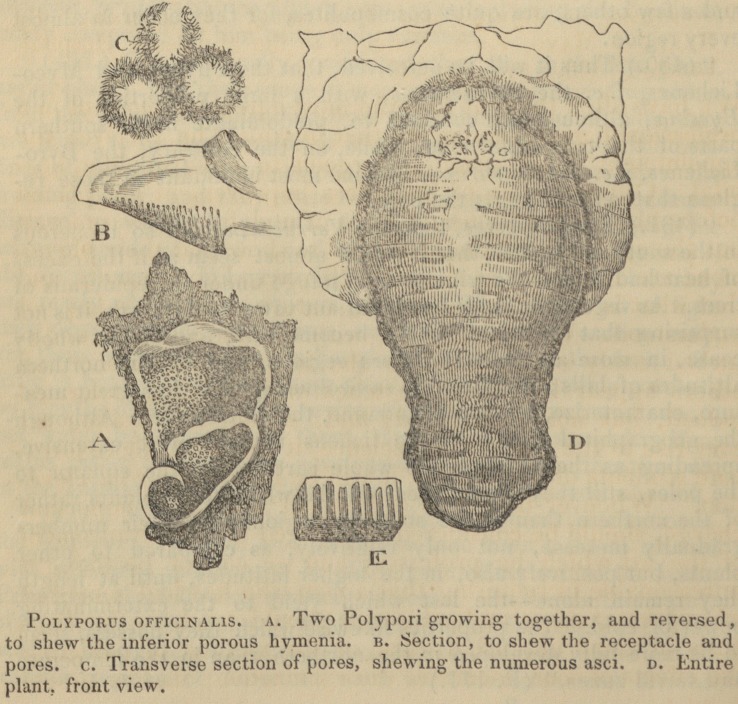# Outlines of Botany; Being a Practical Guide to the Study of Plants

**Published:** 1833-10

**Authors:** 


*Outlines of Botany; being a Practical Guide to the Study of Plants.* By G. T. Burnett, Professor of Botany in King's College, &c. Nos. I. to XI. London. 8vo. pp. 390.

This work differs essentially from any hitherto published on the subject; at least, from all that we have seen. As regards botanical science, the plan may be considered as original; and the advantages of such a scheme being confessed by zoologists, it is only surprising that it has not long since been introduced into botany. In studies so extensive as the several departments of natural history, subdivision is obviously of the utmost importance: hence zoology, botany, &c. were soon distinguished and acknowledged as separate sciences. In zoology further natural subdivisions were speedily introduced; birds, insects, reptiles, and fish, being distinctly studied, and their anatomy, physiology, and habits constituting the materials of the subordinate branches, called respectively ornithology, entomology, herpetology, and ichthyology: and thus the confusion was avoided which would have been the necessary result of studying without such distinctions the structure and the functions of the whole.

In botany, on the contrary, the systematic distribution being rather more artificial, and the great natural groups, although for the most part acknowledged in name, being in general neglected in reality, the study of plants has been considered as a single science, and often thought to be indivisible, because it has not been usual to subdivide it. It is true that partial efforts have been made to distinguish it into subordinate departments, equivalent to the subordinate branches of zoology; but although some groups, as the mosses, fungi, &c., have been occasionally treated of separately, as the subjects of the secondary sciences, muscologia, fungologia, &c., the present is the first work in which this scheme has been attempted to be carried through the whole vegetable kingdom.

The scheme of the work having our approval, the execution must form the next subject of consideration; and we confess that, notwithstanding the favourable prepossessions the plan had excited in our minds, we have read the first eleven numbers with mingled feelings of satisfaction and displeasure, in which, however, the former most decidedly predominate. With the matter we have no fault to find, but the style is peculiar, and formed upon a model long since obsolete. This is chiefly evident in the first fasciculus, which, as we are informed in the preface, was originally delivered as an introductory lecture: but we would suggest to Mr. Burnett, that many sentences flow freely from the speaker's mouth that read uneasily when found in print. For example, take the following passages, which are certainly too abstract for the simple introduction which these Outlines profess to be. It is indeed the bane of science, that the initiated so soon forget the difficulties which they have surmounted.

"(16.) Plants are the subjects of botany; their attributes the objects of the science: hence, two schemes of study, the subjective and the objective, lie before us; each of which may be pursued in opposite courses; *i. e.* either by analysis or synthesis, whence the anterior and posterior arguments result; between these the selection must be made. The former descends from generals to particulars, the latter ascends from effects to causes; that being essentially more abstract, this more practical in its course. Each has advantages peculiarly its own; hence, both should in turn be studied, and neither exclusively neglected or pursued. But, as the anterior argument requires much antecedent knowledge, while the posterior can trace back from none, that being the fruits of learning, while this is the means to learn; although the first is the most comprehensive, the last is the most familiar, and hence it is that with which we shall commence our labours.

"(17.) Although differing essentially from the usual schemes of investigation, synthesis shall here precede analysis, and the subjective now be made the forerunner of the objective view; for it seems advisable, at least occasionally, to commence with a practical demonstration of plants as they are found to exist in nature; and to show their positive characters before comparisons are instituted between them and the other kingdoms of the organic and inorganic worlds: in fact, first to have materials to compare before comparative views are taken. Hence, after giving a general conspective glance at the whole, it is proposed to demonstrate the special structures, functions, properties, and uses of each succeeding group of plants, from the lowest to the highest grades; and this before any general views or comparisons are instituted, even between the varied developments of equivalent organs, as pervading the whole vegetable kingdom, and much before any are made between the different, and often essentially diverse, constitutions of the adjacent animal and inorganic reigns."

Having now discharged the disagreeable part of our duty, which we always like, when possible, to get through first, the path is free for us to discuss those points which have our approbation, and to cull examples to justify our expressed opinion. We shall pass by the introductory sections, and extract the commencement of the General Outline:

"(25.) In the ocean, in rivers, and especially in stagnant water, as well as in many damp situations on shore, myriad of minute animals and plants exist, which for ages were utterly unknown; or, if noticed, were mistaken for the foam of the waves, or the exuviæ of the bodies amongst which they abound.

"(26.) So minute are some of these infinitesimals of vitality, that, in a drop of water, it is said there might be suspended five millions; and eight hundred millions, that is, almost as many as the entire human population of this globe, might, if collected, be contained in the space of one cubic inch.

"(27.) Yet, small as are these *monads*, their structure is by no means so simple as is their bulk reduced; for Ehrenberg describes those species which, from their ultimate atomic minuteness, and resemblance to fine dust, have been called *termo*, *atomus*, and *pulvisculus*, to possess each from four to six, and in the atom many stomachs; and, furthermore, in the allied genera,* he has counted no fewer than from one to two hundred stomachs: *i. e.* from one to two hundred internal sacs, or digesting pouches, into which coloured fluids have been seen to pass; and in many others, these organs are equally elaborate, and the collateral structures curious in the extreme.

"(28.) The most minute vegetables, however, which have been as yet discovered, are much less complex in their structure than animalculæ are found by zoologists to be; for these, in the lowest grades that have been accurately examined, appear to consist of simple cells, or threads, [vid. § 41, 47, 121, &c.] either free or springing from a slimy film, and which, although frequently associated, and often in contiguity, appear, in many cases, to have no necessary connexion with each other.

* Cyclocæla, Orthocæla, Campylocæla, and Paramæcium. From Ehrenberg's monograph on Infusorial animalculæ.

"(29.) Allied to these simplest plants and animalculæ are certain ambiguous beings, which, on the verge of both kingdoms, seem to belong indisputably to neither: for in them some of the most distinctive characteristic signs of animals and vegetables are so conjoined, that at times they would appear to be both, and again indifferently either. Thus, their germs take root and grow like ordinary plants, while the fruit they bear seems to be possessed of voluntary motion, and to pass, in its development, through a stage of animal existence, before it, in its turn, takes root, and bears another generation. *Zoocarpes*, or *fruit animalculæ*, are the names which, not improperly, have been given to these connecting links of the animal and vegetable reigns.

"(30.) Through these neutral tracts, which, while they bound, connect both kingdoms, the oft-disputed line of demarcation runs. From such obscure and debateable beginnings, plants and animals, as the dominion of each is on either side confirmed, gradually become less questionable in their forms, and assume their more essentially diverse structures. At this utmost verge of the vegetable domain, the present demonstration shall commence." (P. 25.)

The nine classes into which Professor Burnett has distributed the vegetable kingdom nearly coincide with those which are popularly acknowledged, and which are admitted by most writers on the subject. They are, 1, the Flags, or Algæ; 2, Mushrooms, or Fungi; 3, Mosses, or Musci; 4, Ferns, or Filices; 5, Grasses and Sedges, or Gramina; 6, Palms and Lilies, the Palmæ and Lilia of Linnæus, forming the Palmares of our author; 7, the Pines and Zamiæ; 8, the Angiospermous Dicotyledonous Plants; 9, the Flowering Cellular Plants, such as the Rafflesia.

In the general outline a short sketch is given of each of these nine groups, the fuller descriptions of which will form the contents of the subsequent fasciculi. We doubt not that others besides ourselves have long been convinced of the necessity of reducing the very numerous natural groups called *orders* or *families* of plants, which, from the fifty-eight of Linnæus, and the hundred of Jussieu, have in modern times been increased to upwards of two hundred and fifty: for their very number has become oppressive, and the orders are themselves in a very confused and disordered state. For example, no two persons agree in their succession, or other disposition, and as little concord is found in their extent; the *Rosaceæ*, *Amentiferæ*, &c. of one writer being five or ten times larger than groups of the same name in another. This, which is the natural result of a progressing science, and the subordinate distribution of the first acknowledged general orders, becomes perplexing to the student, not only from the diversity of extent, but also, and still more so, from the several grades of analysis and synthesis having no signs in their denominations by which they can be distinguished from each other. We know not whether the scheme of nomenclature here proposed will be generally adopted, but it appears to us to have been one of the simplest that could have been devised, and changes as little as possible the names already given to the various groups. The terminations which are now indifferently affixed to the distinctive title of an order, whether of primary, or secondary, or tertiary magnitude and importance, are appropriated by Professor Burnett to the several major and minor groups. Thus, for example, the order *Rosaceæ* in modern systems has been subdivided into the orders *Rosaceæ*, *Pomaceæ*, *Amygdaleæ*, *Dryadeæ*, &c.; the order *Amentiferæ* into the *Cusculiferæ*, *Betulineæ*, *Salicineæ*, &c. Now it is clear that these terminations might easily be made distinctive of larger or smaller groups; just as, in chemical nomenclature, the terminations are distinctive of different acids; *e.g.* the sulphur*ous* and the sulphur*ic*, &c.

As the majority of the orders at present established terminate in *aceæ*, our author proposes that all of them should do so, which may be effected by calling the Butomeæ *Butomaceæ*, the Irideæ *Iridaceæ*, and so on. The larger collective groups, which very frequently, in Professor Burnett's arrangement, almost coincide with the *orders* of Jussieu and Linnæus, are distinguished by the termination *inæ:* thus, the section Acorinæ includes the subsections (or types) Callaceæ, Orontiaceæ, and Lemnaceæ; the section Alisminæ contains the subsections Juncaginaceæ, Alismaceæ, and Butomaceæ; the section Scitaminæ contains the subsections Zingiberaceæ, Marantaceæ, and Musaceæ; and so of the rest. The larger groups, in which the sections again are collected, are known by the names terminating in *ales;* as, for example, the Cyperales.

It would be very easy to find fault with any professedly natural arrangement reduced to such a seemingly artificial state; but, on examination, it will be found that the larger associations are not, in comparison, at all more arbitrary or artificial than the smaller ones, which are the most natural that have been hitherto devised: and thus great simplicity has been given to a subject which has often with justice been considered difficult and complex. This is therefore one of the features in the present work with which we are the most pleased, and which has our hearty commendation; for we must remind our readers, that no such thing as a *really* natural system exists: it is a mere chimera, a beau ideal, which it is well to contemplate in the imagination; but all associations of plants beyond species are more or less arbitrary, and merely conventional assumptions not known to Nature, though essential to her student. Nothing indeed can be more artificial than many genera, which are often dwelt on as being the most natural of all the collective groups, while, on the contrary, they are frequently less so than the more comprehensive classes, orders, and sections in which they are contained: for example, take the generic distribution of the seaweeds, the liverworts, the true mosses, the ferns, the grasses, the sedges, the palms, and other petaloid monocotyledons, or almost any of the larger groups,—who does not recognize the more general affinities, while the subordinate distinctions are frequently faint, and made out with difficulty?

The second fasciculus of Professor Burnett's work contains outlines of Algologia, in which the Algæ, or Flags, *i.e.* the *Confervales*, or articulate flags, the *Fucales*, or inarticulate ones, and the *Lichenales*, or aerial algæ, are treated of in succession. Each of these subdivisions contains an "account of the structure, functions, systematic arrangement, geographical and geological distribution, medicinal, dietetic, and other uses," with historical and critical notices of the plants in question.

Although more collateral knowledge is here condensed than is usually admitted into botanical works, still, from each great natural division of the vegetable world being separately described, the subjects are mastered with much more ease than when dry systematic details form the unvaried topics of *one* work; the anatomy and physiology of the whole vegetable kingdom the subject of another; while the geographical and geological distributions, the uses, &c. of the successive groups are often treated of cursorily, if not altogether disregarded. The following extracts may enable our readers to form some opinion of the manner in which this plan has been executed:

"(264.) DICTYOTACEÆ. The sea networks, forming the first type of this section, are well characterised by the beautifully reticulated texture of the tegument, whence indeed the name *Dictyotaceæ,* which has been given to the group, from its normal genus *Dictyota.* The fronds are of various forms, but in all, excepting *Halyseris,* the sea-endive, ribless; and the conceptacles are pellucid, inclosing the sporules, which are for the most part produced beneath the epenchyme.

"(265.) The Peacock's tail, or *Padina pavonia,* affords a beautiful example of this section; but *Chorda filum,* sea-whiplash, or sea-catgut, is perhaps a more familiar instance. This plant is often found thirty or forty feet in length; and Lightfoot says, the Highlanders twist it, when skinned, into fishing lines: and so abundantly does it sometimes grow, that, as Mr. Neil declares, it is with difficulty a pinnace can make its way through oceanic meadows of this weed.

(*a*) Chorda Filum. (*b*) Portion of frond artificially unrolled to shew its spiral structure. (*c*) Spores magnified. (*d*) Portion in fructification. (*e*) Section, to shew internal structure.

"The frond of this cord-like flag is hollow within, and the channel interrupted at short distances by transverse partitions, the use of which, according to Colonel Stackhouse, is to confine the air, or elastic vapour, to certain spaces; so as to act like swimming bladders and increase the buoyancy of the plant, which extends itself to such an amazing length, and always shoots upwards to the surface.

"(266.) The smell of (Halyseris) the sea-endive, the only genus with a ribbed frond, is said to be, 'when fresh gathered, extremely powerful and disagreeable.'

"(267.) *Chordariaceæ.* The *Chordaria*, or sea-whipcord, which differs from all other Algæ by its *solid* filiform cylindrical frond, even although the fructification is very imperfectly known, has been arranged in a separate section by Greville, who thinks 'its singular structure removes it from all the other orders;' and hence it is the only known example of the *Chordariaceæ*, or twine-wracks.

A. MACROCYSTIS PYRIFERA. B. LAMINARIA BUCCINALIS. (*a*) Transverse section of stem. (*b*) A portion magnified, to shew structure. C. CHORDARIA FLAGELLIFORMIS. (*c*) Transverse section of frond, with fruit. (*d*) Fibres and sporidia. (*e*) Spores still further magnified. (*f*) Longitudinal section of frond magnified.

"(268.) *Sporochnaceæ.* Another type of this section, the *Sporochnaceæ*, which contains the genera *Sporochnus*, or scatter-tuft, *Dichloria*, or changeling, and a genus named in honour of Desmarest, *Desmarestia* or *Desmia*, is chiefly characterised by bearing little tufts of fine green filaments on the fronds, but which are deciduous in some, and not yet observed in all the species. The fructification is collected in tubercles, either stalked or sessile. These plants, which are all marine, and of an olive or yellowish green colour, although they do not change to black in drying, become flaccid on exposure to air, acquiring a verdigris colour, and then possess the curious property of rapidly decomposing other delicate Algæ in contact with them.—*Grev.*

(*a*) SPOROCHNUS PEDUNCULATUS, natural size. (*b*) A receptacle terminated by its tuft of filaments. (*c*) Section of the receptacle. (*d*) Filaments with their fertile summits. (*e*) Portion of a filament of the receptacle.

"(269.) The sea-belts, or sea-girdles (*Laminaria*), the murlins, honey-ware, or bladder-locks (*Alaria*), with the interminable (*Macrocystis*), [§ 267, fig. A.], or bladder-thread, form, with a few other allied genera, such as *Durvillæa*, *Lessonia*, and so forth, a very natural and well-marked type, called, from their flattened

ALARIA ESCULENTA. (*a*) Immature frond. (*b*) Fructiferous leaflet of a mature plant. (*c*) Section, to shew internal structure. (*d*) Spores. (*e*) Filaments issuing from minute pores in the frond. *Grev. Alg.* iv.

form, and from *Laminaria*, or tangle, the name of the normal genus, *Laminaceæ*, or tangle-wracks; by Bory St. Vincent and Greville they are denominated Lamin*arieæ;* this termination, however, as in the other cases where a similar alteration has been made, is only changed from the manifest expediency of designating similar grades of analysis by somewhat similar words.

"(270.) The *Laminaceæ*, or tangles, are all marine, and their structure densely fibro-cellular; the fructification is collected in *sori* on the surface of the frond, which rises from a more or less divided rhizoma, and forms a longer or shorter stipes terminating in a plane expansion, either entire or divided; and sometimes ribbed. These plants are chiefly coriaceous, occasionally membranaceous, and become but little changed in hue on exposure to the air." (P. 115.)

"(424.) The distribution of the Lichenales chiefly assumes a topographical rather than a geographical interest. This will already have become apparent from the notices of stations so frequently introduced, and by which they have been shewn to become such admirable guides in the distinction of some of the officinal barks; and moreover, indexes of the states of their preservation: their general statistics will be found, however, not wholly unworthy of attention.

"(425.) The whole number of known species of this order has been estimated by Fee at between two and three thousand. This, however, is probably too high a sum, even including the Byssinæ, many computed by him being only varieties.

"(426.) Geographically considered, they are, in the first place, aerial plants, and their range is most extensive: proceeding either from the poles, or descending from the polar heights of hills, they are found to be first heralds of life, encroaching even on the confines of perpetual snows, vegetating at a temperature below the freezing point; and they cease not to struggle against every impediment to vegetable growth, for they flourish even among the burning sands of *Africa*, and in the hottest and driest regions of the torrid zone. Wherever light comes Lichens grow, but they are rarely produced in obscure places. When deprived of light they degenerate in their forms, and it is the lowest section only, viz. those approaching to the Fungi, that vegetate in the dark. So little is heat regarded by these plants, that when utterly parched, by months of drought, they revive when rain returns; and even if hot water be poured over them, they are not destroyed. Heat seems rather to favour the development of their fructification, for in the hottest and driest places their apothecia the most abound.

"(427.) With regard to the general geographical distribution of the European Lichens, and no others have been hitherto studied with sufficient minuteness to allow generalizations to be made, Fries gives the following summary account. In the southern parts of Europe, on the shores of the Mediterranean Sea, there are found several species of tropical genera, which likewise occur in the warmer regions of America; such as *Chiodecton* and *Dirina.*

From this southern district, it is believed that other more northern Lichens are absent; such as *Parmelia tartarea*, the *Umbilicariæ*, &c.; while *Evernia villosa*, *Ramalina pusilla*, *Cladonia endiviæfolia*, and many *Parmeliæ*, are present. The *Graphidaceæ* are also here abundant.

"(428.) Along the whole western coasts of the Atlantic, even from the south of Spain to Finmark, many of the same Lichens are common; such as Ramalina scopulorum, and various Strictæ and Parmeliæ: the moist atmosphere and more agreeable temperature of a maritime station favouring the extended range. This tract, however, may be subdivided into northern and southern regions: in the latter, the *Roccella tinctoria*, *Sagedia aggregata*, and numerous *Verrucariæ* and *Graphidaceæ* are found; in the former, *Parmelia gelida*, *Biatora atrorufa*, and the *Umbilicariæ*, predominate.

"(429.) In the Arctic regions, as in Iceland, and especially in the alpine parts of Lapland, the *Cetrariæ* and *Cladoniæ* prevail: the former flourishing on the tufa and volcanic scoriæ; the latter clothing an otherwise barren soil, even from the sea-shore to the summits of the mountains. In these districts, *Evernia vulpina*, and many other lichens, cease to grow; as the Calicia do in warmer regions: for Fries observes, that in the tropics these last-named lichens are unknown. *Usnea barbata* and *Cladonia pyxidata*, and a few others, are quite cosmopolites, for they occur in almost every region.

"(430.) Thus it will be perceived, that the Phyco- and Myco-Lichenes, *i. e.* the *Verrucarinæ*, with a large proportion of the *Byssinæ*, although not confined to, predominate in the southern parts of the temperate zone; while, on the contrary, the Byro-Lichenes, *i. e.* the *Cetrarinæ*, become most abundant in those regions that verge towards the pole.

"(431.) Fries observes, that the *Verrucarinæ* are so numerous in the southern regions, that it would almost seem as if the excess of heat had driven the tribe to take refuge under the epidermis of trees. As vegetation is far less luxuriant towards the north, it is not surprising that epiphytic lichens become rare, and at last wholly cease, in more and more northern regions, and on the northern altitudes of hills; and that the saxicolous species, in a great measure, characterize, by their abundance, the arctic zone. Although the geographical range of the lichens is thus most extensive, spreading as they do over the whole earth, from the equator to the poles, still they should seem on the whole to be plants rather of the northern than of the southern regions; for their numbers gradually increase, not only relatively, as compared to other plants, but positively also, in the higher latitudes, until at length they remain alone—the last which yield to the exterminating power of cold. The properties likewise which they possess, seem to be more fully developed in the northern than in the temperate and torrid zones." (P. 171.)

"(668.) Several of the Polypori are possessed of more or less important medicinal virtues. *Polyporus igniarius* has long been famed as a styptic; *P. annosus* is reported by the Swedish peasantry to be a cure for snake-bites; and *P. officinalis* is enumerated by the Germans as one of the articles in their extensive list of vegetable medicines: its action is cathartic. Amadou, or German tinder, is made from the *P. igniarius*, by separating the porous hymenium from the harder parts, and steeping it in a solution of nitre, after it has been beaten into a soft and spongy state. Various other species of Polyporus, besides the igniarius, as the *hispidus*, &c., retain fire when dry, and are also collected and used as amadou. The Laplanders have long been in the habit of employing these, and other fungi, for the same purpose, and in a similar way, as the natives of Japan and China do the moxa. Whenever they suffer from pains in their limbs, they bruise some of the dried fungus, or amadou, and, pulling it to pieces, put a small heap of it on the part nearest to the seat of the pain. It is then set on fire, and, burning away, it blisters the skin; and, although some persons may think it a coarse and rough method of treatment, it is generally a very successful one. *Polyporus suaveolens* has a smell like that of aniseed, and it is one of the few luxuries of Lapland. Linnæus says that the odour is there so much admired, that the young men carry it

POLYPORUS OFFICINALIS. A. Two Polypori growing together, and reversed, to shew the inferior porous hymenia. B. Section, to shew the receptacle and pores. C. Transverse section of pores, shewing the numerous asci. D. Entire plant, front view.

about them when they visit their mistresses, in order to render themselves more agreeable.

"(669.) It is not unlikely that other species of Polypore may possess useful properties, or might be resorted to as the sources of valuable drugs. From *P. dryadeus* (the old *Boletus pseudo-ignarius*), Braconnet obtained his boletic, and from *P. squamosus* his fungic acid; and from *P. sulphureus* Dr. Scot, of Dublin, and Drs. Greville and Thompson, of Edinburgh, have procured oxalic acid and bin-oxalate of potash. Mr. Purton had previously noticed the pungent acid taste of this fungus, and especially of the porous part; and I once found an enormous mass of it, like that described by Dr. Greville, on an old willow-trunk in Kensington Gardens, which, while drying, became covered thickly, as if frosted over with a white salt, the bin-oxalate of potash, some of which, with part of the fungus, I now have by me." (P. 248.)

The woodcuts with which Professor Burnett's book is illustrated, and with several of which the publisher's kindness has allowed us to adorn our pages, will most materially facilitate the progress of the young botanist. In a word, the work is an excellent one, and deserves, not a superficial perusal, but the deepest study.

## Figures and Tables

**Figure f1:**
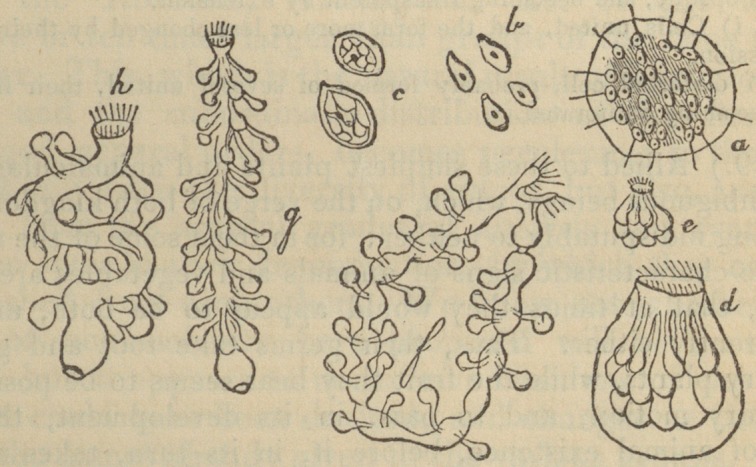


**Figure f2:**
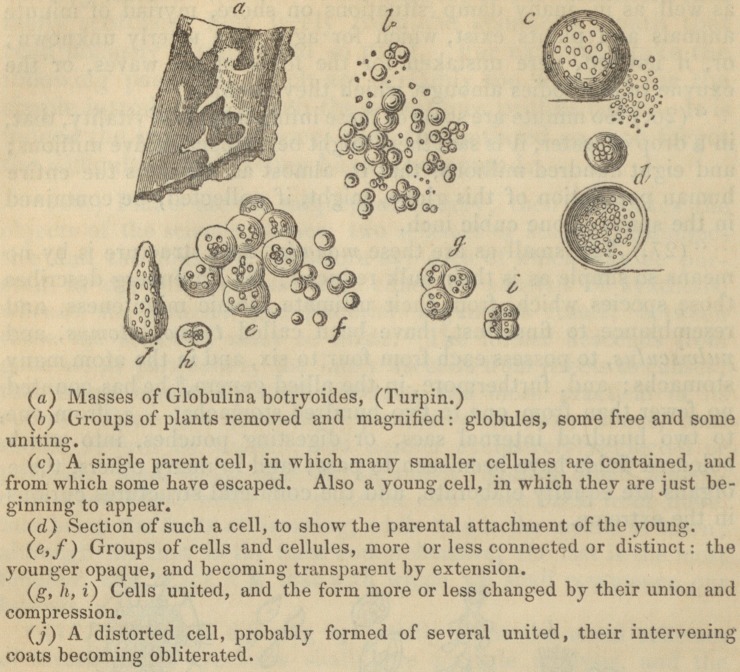


**Figure f3:**
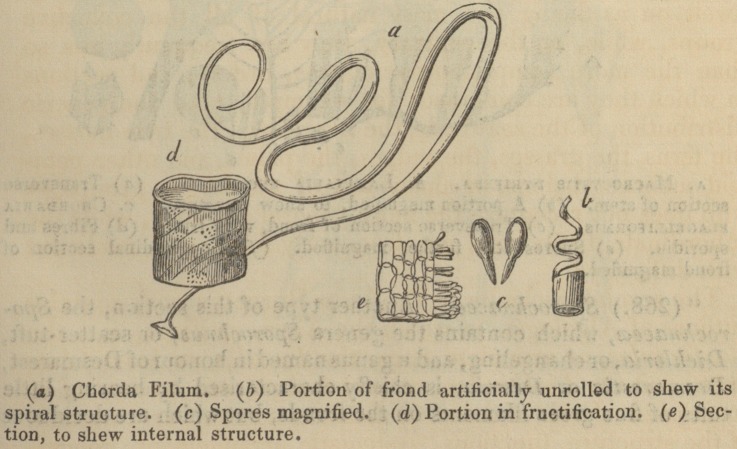


**Figure f4:**
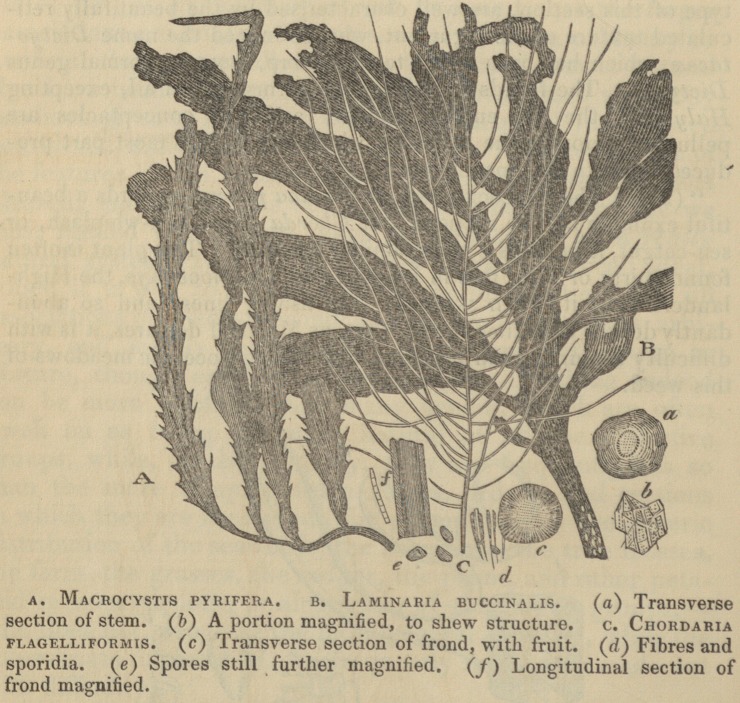


**Figure f5:**
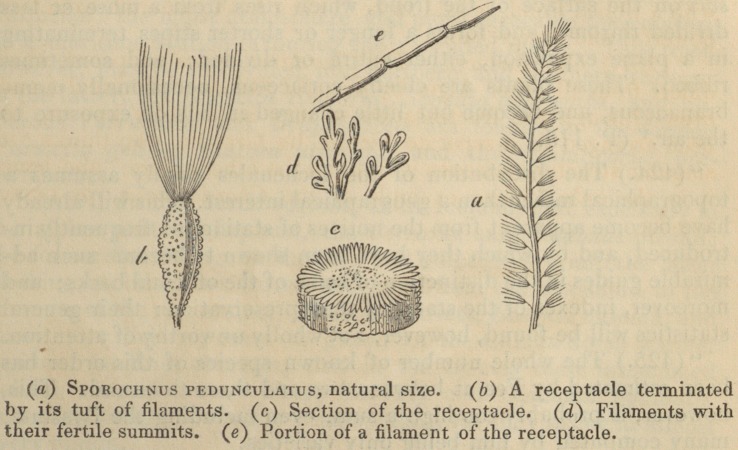


**Figure f6:**
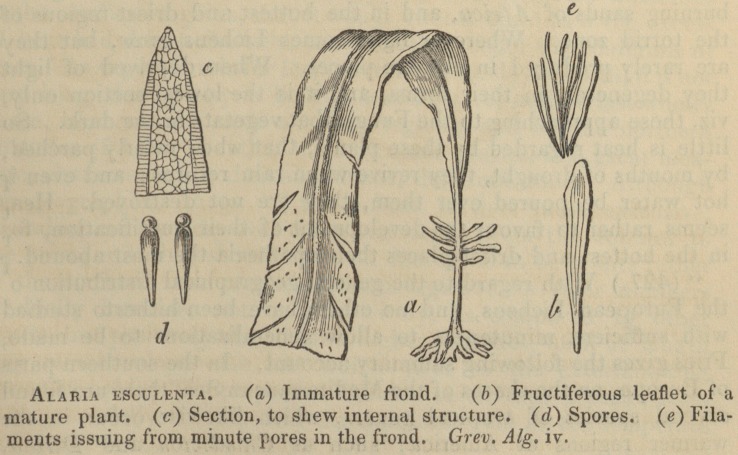


**Figure f7:**